# *Microcystis* plasmids: the unexplored portion of the mobilome and the presence of potential phage-like plasmids

**DOI:** 10.1093/ismeco/ycaf154

**Published:** 2025-09-03

**Authors:** Gwendolyn F Stark, Laura E Smith, Alexander R Truchon, Robbie M Martin, Elizabeth R Denison, Steven W Wilhelm

**Affiliations:** Department of Microbiology, The University of Tennessee, Knoxville, TN 37996, United States; Department of Microbiology, The University of Tennessee, Knoxville, TN 37996, United States; Department of Microbiology, The University of Tennessee, Knoxville, TN 37996, United States; Department of Microbiology, The University of Tennessee, Knoxville, TN 37996, United States; Department of Microbiology, The University of Tennessee, Knoxville, TN 37996, United States; Department of Microbiology, The University of Tennessee, Knoxville, TN 37996, United States

**Keywords:** phage-like, cyanobacterial blooms, mobile genetic elements, horizontal gene transfer

## Abstract

While resequencing *Microcystis aeruginosa* (PCC7806) and its nontoxigenic mutant (PCC7806 Δ*mcyB*), we discovered identical unreported plasmids in both strains. These strains were separated in culture over 25 years ago, resulting in sequence divergence among their chromosomes. RNA-seq data demonstrated these plasmids were transcriptionally active during chemostat growth. Moreover, *in situ* metatranscriptomes from Lake Erie revealed genes like those on the PCC7806 plasmid were expressed in the environment. As we investigated plasmids in *Microcystis*, we found that *M. aeruginosa* NIES-298 also had a putatively conserved plasmid, but with phage-like features. To gain an understanding of the ecological relevance of these plasmids, we examined Lake Erie metatranscriptomes and found that transcript abundance for predicted plasmid-like contigs was significantly higher than predicted virus-like contigs across the microbial community: this trend was also present when metatranscriptomic reads were mapped to *Microcystis*-infecting phage and *Microcystis*-specific plasmid genomes. Our observations demonstrate a potential ecological importance and stability of these extrachromosomal elements in *Microcystis*. Additionally, this work draws attention to the potential overlap between *Microcystis* plasmid and phage genomes, and how this may complicate molecular investigations.

## Introduction

*Microcystis aeruginosa* is a well-studied cyanobacterium, yet there remain aspects of its physiology and ecology that are poorly understood. One overlooked feature of *Microcystis* spp. are their plasmids. Historically, physiological, and ecological studies have ignored *Microcystis* spp. plasmids almost completely. In the 1980’s, before microcystin production was determined to be chromosomally encoded [1], it was thought that plasmids might play a role in its biosynthesis [2]. Early research on *Microcystis* plasmids using southern hybridization techniques showed that cells can harbor multiple plasmids, that plasmid genes can share homology with host chromosomes, and that identical plasmids were found in geographically isolated *Microcystis* [3]. This early research provided insights into *Microcystis* plasmid structure and conservation. However, since that time there has been limited research on *Microcystis* plasmids and their physiological and ecological importance [3–5].

While plasmids have been overlooked, phages that infect bloom-forming cyanobacteria have been better-studied. Originally examined as possible biocontrol agents for blooms [6], researchers more recently have become interested in how phage might transform cyanotoxins in terms of size class distribution–shifting toxins from the particulate to dissolved phases in aquatic systems [7]. While phages are clearly important mobile components of harmful algal blooms (HABs), there are other infective mobile elements, known as phage-plasmids, which have not been explored in HAB datasets. Phage-plasmids are of importance, as they are not only capable of producing infective particles, but also exist as plasmids and promote recombination between phage and plasmid elements [8]. Yet depending on the methods used in metagenomic analyses, classification of these elements may be obscured and not properly accounted for, much like *Microcystis* plasmids.

We recently re-sequenced *M. aeruginosa* PCC7806 as well as its nontoxic, genetically engineered mutant, *M. aeruginosa* PCC7806 Δ*mcyB* [9], due to our interests in genomic rearrangements and the long history of these strains in lab studies [1, 10–19]. These strains have been “genomically-isolated” since the mutant was created in 1997 [1], which has been long enough that the chromosomal genomes have diverged [20]. During this process we found the DNA sequence of the plasmids remained completely conserved, contrasting the divergence seen in the chromosomal genomes. Following this observation, we expanded our analyses to include *M. aeruginosa* NIES-298, the host for *Microcystis* phage Ma-LMM01 [21]. This strain was previously known to contain a plasmid, which we now show has phage-like features, and may also be conserved at the DNA sequence level. Our NIES-298 isolate also contained a second, previously unreported plasmid, which contained transposable elements that displayed minor sequence variance across in-lab isolates. To understand the ecological relevance of plasmids in HABs, we examined metatranscriptomes from a Lake Erie *Microcystis* bloom to investigate the activity of all plasmid-like contigs as well as *Microcystis*-specific plasmids relative to all virus-like contigs and *Microcystis*-specific phage. Collectively we demonstrate the conserved nature of *Microcystis* plasmids, their potential functionality, and how relevant information from HAB datasets may be lost by not considering plasmids. Moreover, we touch on the overlap between *Microcystis* plasmid and phage genomes, and how genetic similarities may complicate HAB ecology research using specific gene markers.

## Methods and materials

### *Microcystis* cultures, growth conditions, and DNA extraction

The strains used in this study were *M. aeruginosa* PCC7806 wildtype (accession #CP155078), *M. aeruginosa* PCC7806 Δ*mcyB* (GCA_030553035.1), and *M. aeruginosa* NIES-298 (GCA_048820285.1). Information on PCC7806 and Δ*mcyB* origins, strain history in our lab, and growth conditions have been detailed in Stark *et al.* 2023 [22]. In brief, strains were grown in 25 ml of modified CT media at 26° C and ~35 μmol photons m^−2^ s^−1^ under a 14-h/10-h day/night cycle. *M. aeruginosa* NIES-298 is available from the *National Institute for Environmental Studies Microbial Culture Collection* (Japan). The NIES-298 wild-type strain is susceptible to infection by the *Microcystis* phage Ma-LMM01, which we received as a kind gift from Professor Takashi Yoshida (Kyoto University). As part of a parallel study, we isolated, grew, and sequenced three lab-derived phage-resistant (Ma-LMM01) isolates (generated in-house and denoted “NIES-298B”, “NIES-298C”, and “NIES-298D”). Mutant strains were generated from clones that resisted lysis after viral exposure in our lab—these mutants are available upon request. Growth conditions were the same as those for PCC7806 strains. For all strains a high molecular weight DNA extraction protocol was employed [9].

### DNA sequencing, assembly, and annotation

We re-sequenced the genomes of *M. aeruginosa* PCC7806, and *M. aeruginosa* PCC7806 Δ*mcyB* [20] as well as wildtype and phage-resistant mutants (B, C, and D) of *M. aeruginosa* NIES-298. Library preparation, high-molecular weight DNA extraction and both long-read (in-house Nanopore) and short-read (Illumina, at SeqCenter, Pittsburgh, PA) sequencing were completed as previously reported [9]. Plasmids were assembled alongside genome assemblies using Unicycler (v.0.4.9b) [23], and detailed hybrid assembly methods can be found in Stark *et al.* 2023 [9]. All plasmids were annotated using NCBI’s prokaryotic genome annotation pipeline (PGAP) [24].

### Identifying PCC7806- and NIES-298-like plasmids in other draft genome assemblies

A nucleotide BLAST search (highly similar sequences) of the PCC7806 (accession PV068374) and NIES-298 plasmid sequences (NIES-298 has two plasmids that we refer to as “P1” (accession PV068373) and “P2” (accession PV076616) was conducted against *Microcystis* scaffold-level genomes (taxid:1125) in the whole genome sequencing database (WGS). There were 341 scaffold-level *Microcystis* genomes as of access date April 2024. Contigs from genomes that had 100% query, and 100% identity were considered identical. Hits ≥30% query cover and ≥90% nucleotide identity were recorded. Contig hits that met our BLAST parameters were analyzed using geNomad, with default settings [25], to obtain plasmid, virus, and chromosome scores. Unless noted, all search parameters were kept as default.

### Comparing PCC7806 and NIES-298 plasmids to their respective host genomes

To identify any similarities between plasmids and their host genomes (a hallmark of putative gene flow), a BLAST search of the PCC7806 and NIES-298 plasmids was done against their respective host genomes. In the case of the NIES-298 plasmids, we also did a BLAST search against the genome for *Microcystis* phage Ma-LMM01, which can infect wildtype NIES-298 [21]. An e-value was set to 1E-05, and all other parameters were kept as default. BLAST searches were visualized using Proksee [26].

### Calculating the PCC7806 plasmid copy number

To estimate *M. aeruginosa* PCC7806 plasmid copy number, we used Illumina short-reads to determine coverage of the entire Δ*mcyB* and wildtype genomes. Reads were paired and trimmed using CLC genomics workbench (v.21.0.4). For genome mapping, paired and trimmed reads from *M. aeruginosa* PCC7806 Δ*mcyB* (5,103,923 bp) or the wildtype (5,096,229 bp) were mapped to their respective genomic assemblies and the plasmid (8,505 bp) using CLC genomics workbench (v.21.0.4), using a length fraction of 0.9 and similarity fraction of 0.9, and all other parameters were kept default. Average coverage of the genome and the plasmid were calculated for each sequencing set. To estimate copy number, the average coverage of the plasmid was divided by the average coverage of each respective genome (PCC7806 wildtype and Δ*mcyB*).

### Protein structure models and homology

Colabfold [27] was used to generate protein structure models for the predicted genes from the *M. aeruginosa* PCC7806 plasmid. We also used this tool to model genes from the *M. aeruginosa* NIES-298 P1 plasmid. Protein input sequences were derived from PGAP annotations for all the genes. For all runs, the PDB100 template was used. The number of recycles was set to six and the number of structures to relax was set to five. All other settings were kept as default. To visualize protein structures, NCBI iCn3D 3D-structure viewer was used [28, 29]. To find putative predicted functions for the PCC7806 plasmid genes and NIES-298 P1 plasmid genes that had no homology to known protein domains on PFAM or HMMER [30], we ran our ColabFold structures through DALI [31]. The ColabFold models submitted to DALI were the relaxed fit, rank 1 structure for each protein. Models were run against the whole PDB database. Structures with pLDDT values >70 were considered for protein structure predictions. The top result (highest Z-score) was recorded for each protein model, and all Z-scores >2 were considered significant [31].

### RNA-seq analyses of PCC7806 plasmid in culture

For gene expression analysis of the PCC7806 plasmid, we used transcriptomic libraries generated during an experiment that examined temperature effects on microcystin production and gene expression [9]. Transcriptomic libraries were generated from axenic monoculture chemostats for both the PCC7806 wildtype and Δ*mcyB* mutant (*n* = 2 independent chemostats each). All transcriptomic libraries were paired-end and trimmed for quality using CLC Genomics (v.21.0.4) as described in Stark *et al.* 2023 [32]. To gauge plasmid gene expression relative to chromosomal genes in PCC7806, reads were mapped to a concatenated assembly of the *M. aeruginosa* PCC7806 wildtype genome (accession CP155078), and the PCC7806-plasmid using CLC Genomics workbench (v. 23.0.4). Mapping parameters and *in silico* ribosomal reduction for transcriptomic reads were completed as previously described [32]. GraphPad Prism (v.10.2.2) was used to visualize and compare transcript mappings, which were normalized for each library by “transcripts per million” reads (TPM). This metric normalizes for both sequencing depth (total number of reads) and transcript length, providing a relative measure of expression.

### Sample collection and design for Lake Erie nutrient-amended microcosm metatranscriptomes

To investigate the ecological relevance of whole-community and *Microcystis*-specific plasmids, we used metatranscriptomes from two independent microcosm experiments previously conducted on *Microcystis*-dominated communities in Lake Erie [33, 34]. The microcosm co-assembly published in Pound *et al.* 2022 [33] will be referred to as “Experiment 1” (denoted E1) and the microcosm co-assembly published in Martin *et al.* 2023 [34] as “Experiment 2” (denoted E2). Detailed information regarding metadata and sample collection for both studies has also been published [34]. For the present manuscript, we only used the metatranscriptomes derived from control bottles (no nutrient addition). Each treatment had *n* = 3 true replicates. RNA samples were collected at 48 h and processed as previously described [34].

### Metatranscriptome analyses of the PCC7806-like genomes and plasmids in Lake Erie

To determine whether genes with high similarity to those on the PCC7806 plasmid were transcribed in Lake Erie, we queried the control metatranscriptomes from E1 and E2. All metatranscriptome libraries were previously trimmed, paired, and had *in silico* ribosomal reduction completed [34]. For recruitment, we used the concatenated genome assembly of the PCC7806 wildtype genome and plasmid (see above). Transcriptomic reads were mapped as described above, using stringent mapping parameters (0.9 length, 0.9 similarity fractions). The maximum number of different hits for a read was set to 30, all reads were normalized to TPM, and paired reads were counted as one.

### Identifying putative virus- and plasmid-like contigs in Lake Erie metatranscriptomes

Separate co-assemblies were previously created for E1 and E2 from the Lake Erie microcosm metatranscriptome experiments [33, 34]. Briefly, reads were trimmed for quality using CLC Genomics workbench (v.20.0.4), and an *in silico* ribosomal reduction was done using SortMeRNA (c. 4.2.0). All nonribosomal reads were assembled into co-assemblies using MegaHit (v.1.2.9). To determine whether any contigs were virus or plasmid, contigs ≥1000 bp were examined with geNomad [25]. For geNomad runs, enable-calibration was turned on and all other settings were set as default. geNomad provides a probability score (out of 1) of how likely a contig is to be “chromosome”, “virus”, or “plasmid” based on marker genes and nonlocal sequence motifs. From the aggregated calibrated score output list for the E1 and E2 co-assemblies, contigs with chromosome scores >0.2 were filtered out, to conservatively focus on contigs that were either phage-like, plasmid-like, or which fell in-between those categories, being neither exclusively more phage nor plasmid-like. Contigs were assigned to “plasmid-like” or “virus-like” categories if the plasmid or virus aggregated calibrated scores were ≥0.7, respectively. We used calibrated probability scores of 0.7 as the minimum cutoff for these categories, which is the geNomad default when run non conservatively. Contigs that fell outside that range (chromosome score <0.2 and plasmid-score and virus-scores <0.7) were labeled as “ambiguously-scored”. Contigs were annotated using geNomad.tsv (converted to .gff) output gene files.

### Relative transcript abundance of putative virus- and plasmid-like contigs in Lake Erie metatranscriptomes

Whole contig coverage was determined for our subset of geNomad-identified contigs from E1 and E2 to define relative transcript abundance of the virus-like, plasmid–like, and ambiguous contigs in the control microcosm metatranscriptome libraries. To calculate contig coverage, CoverM (v.0.7.0) was used (https://github.com/wwood/CoverM). Minimum sequence identity was set to 95% and length fraction was set to 90%, and average trimmed mean (removes outlier regions to calculate the mean coverage of a contig) was used to determine coverage for all individual contigs. From this output, we calculated the average relative transcript abundance, calculated as the proportion of total reads that mapped to our virus-, plasmid-like, and ambiguous contig groupings. Additionally, average coverage per contig for each of our three groupings was also calculated using the CoverM output. Average coverage per contig was calculated by summing up the trimmed mean contig coverage outputs for each group (virus, plasmid, and ambiguous) and dividing by total contigs in each respective grouping.

### RNA-seq analysis of putative virus- and plasmid-like contigs in Lake Erie

As a follow up to our contig coverage calculations, we used geNomad to annotate our contig groupings in the E1 and E2 co-assemblies and examined normalized transcripts that mapped only to predicted gene regions using the same control metatranscriptome libraries. Libraries were normalized by TPM mapped reads. We then found the average TPM per gene for our virus-, plasmid-like and, ambiguous contig categories, by summing up TPM for each group and dividing by the total amount of genes in each group. For transcript mapping, we used a length fraction of 0.9, a similarity fraction of 0.95, and maximum different hits per read was set to 30. Paired reads were counted as one.

As our original groupings of our contigs were more relaxed (calibrated scores ≥0.7), we re-ran the RNA-seq analyses using a more conservative grouping of contigs to see if the same patterns were upheld for our plasmid and virus-like contig categories. For a more stringent analysis of gene mappings, we restricted our analysis to plasmid and virus contigs with scores ≥0.9 (90% chance of being viral or plasmid) in the geNomad plasmid and virus output lists. RNA-seq analysis was done using CLC Genomics Workbench (0.9 length fraction, 0.95 similarity fraction, max hits per read 30).

### Lake Erie metatranscriptomic mappings using known *Microcystis*-specific phage and plasmids

To focus on the gene activity of *Microcystis*-specific plasmids relative to *Microcystis*-specific phage in Lake Erie, we again used the metatranscriptome libraries from E1 and E2, and completed an RNA-seq analysis using CLC Genomics Workbench (v. 23.0.4) (settings: 0.9 length fraction, 0.95 similarity fraction, max hits per read 30) on a concatenated assembly of three complete *Microcystis*-specific phage genomes, and the ten *Microcystis* plasmid genomes available on NCBI GenBank, including the plasmid from PCC7806 (See Supplementary Tables). Libraries were normalized by TPM, and average TPM per gene (per genome) was calculated.

## Results

### *Microcystis aeruginosa* PCC7806 isolates have a conserved plasmid

We uncovered identical 8,505-bp circular plasmids in both our *M. aeruginosa* PCC7806 wildtype and Δ*mcyB* sequencing datasets. Plasmid copy number was estimated as ~15 for both the wildtype PCC7806 (15.1 copies per cell) and Δ*mcyB* (14.8 copies per cell). A BLASTn search using the PCC7806 plasmid as query showed a nearly identical match (98% query, 100% nucleotide identity) to an 8,373-nt long contig (contig 272, accession AM778902) from a 2007 draft version of the *M. aeruginosa* PCC7806 genome (Table S2) [35], confirming the plasmid was not acquired in our lab. Putative protein annotations for the PCC7806 plasmid, using DALI and PFAM, are presented in Fig. S1 and Tables S3 and S4. These predicted proteins included a putative seryl-tRNA synthetase (gene 1), transcription/translation factors (genes 3,4) and elements potentially involved in horizontal gene transfer: a tyrosine recombinase (gene 6) and a Mu-type HTH domain (gene 8).

### Other *Microcystis* spp. have identical or similar plasmids to PCC 7806

Our searches determined that other *Microcystis* spp. harbored identical or similar plasmids to the PCC7806 plasmid. The draft genomes of four geographically and phylogenetically distant *Microcystis* spp. harbored contigs identical or similar in nucleotide sequence to the PCC7806 plasmid (Table 1, Table S5). Three of these contigs were classified as plasmids by geNomad prediction (Table S5). Two of these contigs, from *M. aeruginosa* Ma_AC_P_19900807_S300 and *M. aeruginosa* LEGE 91341, had direct terminal repeats at the contig ends, signifying putatively closed plasmid sequences (Table S5). This indicates that plasmids identical or closely related to the PCC7806 plasmid are harbored by strains of *Microcystis* from distant geographic regions.

**Table 1:** Output of BLASTn for the PCC7806 plasmid nucleotide sequence against the *Microcystis* spp. shotgun genomes.

Description	Query cover	Percent identity	E-value	geNomad plasmid score	Country isolated
*M. aeruginosa* Ma_AC_P_19900807_S300	100	100	0	0.9417	Canada
*M. aeruginosa* PMC 728.11	81	99.88	0	0.9457	France
*Microcystis* sp. CTOTU1208	71	90.82	0	0.1504	Missing (ERX682739)
*M. aeruginosa* LEGE 91341	32	94.43	0	0.956	Portugal

### Predicted genes of the PCC7806 plasmid are expressed in lab cultures and in Lake Erie

**Figure 1:**
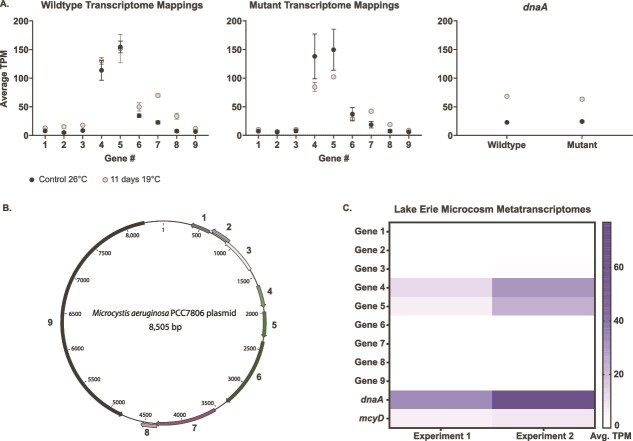
(A) Average normalized transcript mappings reported in TPM (transcripts per million mapped reads) of the nine plasmid genes from the PCC 7806 wildtype transcriptome chemostat read mappings (far left), and average TPM of the nine plasmid genes from the mutant Δ*mcyB* chemostat transcriptome read mappings (middle). As a comparison, we also show the average TPM of the chromosome replication protein gene, *dnaA*, from the PCC 7806 wildtype genome. The transcriptomes were previously generated during a chemostat temperature experiment (*n* = 2), during growth at 19° C and 26° C. The *M. aeruginosa* PCC 7806 wildtype genome assembly was concatenated with the PCC 7806 plasmid, which transcriptomic libraries were mapped to and normalized by TPM. For ΔmcyB transcriptomes, average TPM for gene 4 was 84.3 (± 8.02) at 19° C, and 137.9 (± 0.19) at 26° C. The average TPM for gene 5 was 86.7 (± 2.19) at 19° C and 133.2 (± 32.99) at 26° C. For the wildtype transcriptomes, gene 4 had an average TPM of 130.3(± 5.94) at 19° C and 113.7 (± 17.4) at 26° C. For gene 5, average TPM was 134.4 (± 20.24) at 19° C and 133.9 (± 7.18) at 26° C. A two-way ANOVA (Geisser-Greenhouse and Dunnett’s test correction for multiple comparisons) determined gene 4 and gene 5 were not significantly different (*P* > .05) from dnaA at 19° C, but were significantly higher than *dnaA* at 26° C. For Δ*mcyB* transcriptomes, *dnaA* had average an TPM of 24.3 (± 1.52) at 26° C, and 63.4 (± 3.10) at 19° C. For the wildtype transcriptomes *dnaA* had an average TPM of 22.9 (± 1.37) at 26° C, and 68.4 (± 1.51) at 19° C. (​B) Image of the PCC 7806 plasmid with PGAP generated predicted genes. Genes are numbered 1–9, which correspond to the gene numbers in A and C. (C) Heatmap showing the average TPM of the nine PCC 7806 plasmid genes based on mappings from Lake Erie microcosm metatranscriptomes (*n* = 3) generated by Pound *et al.* in 2022 (experiment 1) [33] and Martin *et al.* in 2023 (experiment 2) [34]. The average TPM of the PCC7806 plasmid genes was compared to the average TPM of *dnaA* and *mcyD* in the *M. aeruginosa* PCC 7806 genome.

We analyzed time-series transcriptomes from an in-lab temperature shift chemostat experiment and observed expression from all nine PCC7806 plasmid genes. Gene expression patterns between the mutant and wildtype were similar. We found that PCC7806 plasmid genes 4 and 5 were the most highly expressed genes on the plasmid (Fig. 1A, Table S6). At 26° C, genes 4 and 5 reached expression levels ~5× that of *dnaA*, a gene encoding a chromosomal replication protein in the PCC7806 genome, and at 19° C, they were roughly double that of *dnaA* (Fig. 1A, Table S6).

We also searched for the PCC7806 plasmid sequences in the Lake Erie microcosm metatranscriptomes from E1 and E2, which showed evidence of transcripts mapping to some genes on the PCC7806 plasmid (Fig. 1C). Like lab strain observations, transcripts were highly recruited to PCC7806 plasmid genes 4 and 5 *in situ* for both E1 and E2 (Fig. 1C, Table S7). Genes 4 and 5 were as or more represented than microcystin-toxin marker gene, *mcyD*, from PCC7806, but not *dnaA* (Table S8). Further output of the RNA-seq and statistical analyses can be found in Tables S7 and S8. Contigs with similar gene content and synteny to the PCC7806 plasmid were also identified in the E2 co-assembly (Fig. S2). This indicates that similar plasmids to PCC7806 may be present and active in Lake Erie communities.

### Plasmids found in other *M. aeruginosa* strains

As part of a separate research effort, we re-sequenced the genome of *M. aeruginosa* NIES-298 and three virus-resistant mutants generated in our lab. We found two plasmids in each genome, with one of the plasmids (which we refer to as NIES-298 P1) being conserved at the DNA level in each of the four genome assemblies. NIES-298 P1 is 18,380-bp in length and has nearly perfect nucleotide identity to a 2019 plasmid sequence from a culture of NIES-298 (accession CP046059.1), albeit that plasmid sequence is 1108 bp shorter (Fig. S3). NIES-298 P1 was also described as contigs in two other draft genomes of the NIES-298 genome, both of which were sequenced in 2017 (accessions BEIU01000026.1 and BEYQ01000022.1) (Fig. S3). These contigs were identical to our NIES-298 P1 (100% nucleotide identity), aside from direct terminal repeats at the end of each contig, signifying putatively closed circular plasmids.

### Conserved plasmid P1 in NIES-298 is phage-like

**Figure 2:**
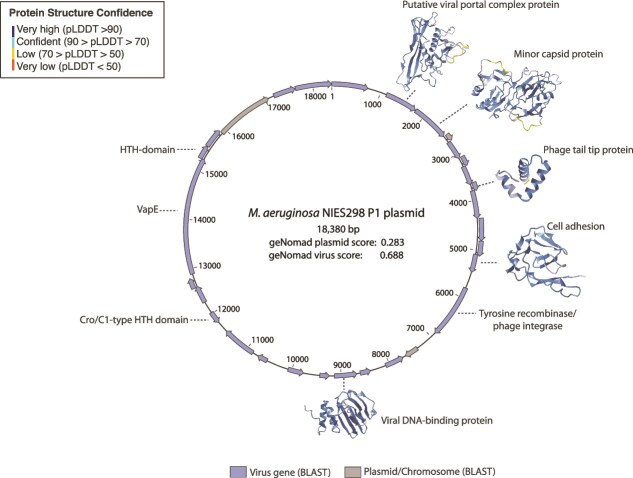
Plasmid map of the NIES-298 P1 plasmid, with genes color coded based on the nucleotide BLAST search result of each gene. If a bacteriophage was the top hit for the gene, it is colored blue. If the top hit for the gene was not bacteriophage, then the gene is colored grey, for more details, see Table S10. All but three genes for the NIES-298 P1 plasmid had sequence similarity to uncultured bacteriophage contigs. The putative viral portal complex protein shared the same Z-score as the top hit, an HMG-CoA enzyme, which is why we putatively assigned it as a viral portal protein

geNomad and BLASTn searches revealed NIES-298 P1 had sequence similarities to bacteriophage (Fig. 2, Table 2, S9, S10). The geNomad score was 0.283 for plasmid and 0.6876 for virus (Table S1). Genes on NIES-298 P1 had significant BLAST hits to uncultured phage contigs or *Microcystis*-specific phage (Table 2, Table S10). Based on protein structure model homology, phage-like genes on P1 included a putative phage tail tip protein (conserved in some *Microcystis* phage genomes), a putative minor capsid protein, and a DNA-binding protein (Fig. 2, Table S11).

**Table 2:** NCBI BLASTn “highly similar sequence” search results for our NIES-298 P1 plasmid sequence.

Description	Query cover (%)	Percent identity	E-value	Accession
*M. aeruginosa* NIES-298 unnamed plasmid 1	94	100	0	CP046059.1
Uncultured phage DNA, contig: NODE385	71	87.02	0	LC425520.1
Bacteriophage sp. contig_8960	43	87.91	0	MT840190.1
*M. aeruginosa* NIES-298 chromosome	4	93.48	0	CP046058.1
Bacteriophage sp. joined_contig_348	7	93.72	0	MT840189.1

P1 shared little sequence homology with either the NIES-298 chromosome or the genome of *Microcystis* phage Ma-LMM01. Our BLAST hits for all three genomes contained sequences similar to CRISPR spacer repeat regions in the *M. aeruginosa* NIES-298 host chromosome (Fig. S4 and S5). Additionally, a tyrosine recombinase/phage integrase on the P1 plasmid shared sequence similarity to an area adjacent to a CRISPR spacer repeat region in the NIES-298 genome (Fig. S6).

### NIES-298 contained a second plasmid

We also identified a second plasmid in NIES-298 that was previously unreported. This plasmid is 43,344 bp in size, and encodes forty-seven putative genes, forty of which have protein coding potential. We refer to this plasmid as NIES-298 P2. We found a nearly exact copy (99% identity) of our P2 plasmid annotated as part of a closed chromosome from a culture of *M. aeruginosa* NIES-298 whose genome was published in 2019 (accession CP046058.1). We are unsure if this is due to assembly error (due to numerous highly repetitive sequences) or if the plasmid is capable of integration. However, for our four hybrid assemblies, NIES-298 P2 consistently assembled as a circular extrachromosomal contig, leading us to designate it as a second plasmid.

While NIES-298 P1 remained the same between our sequencing runs, P2’s sequences varied slightly. P2 has 17 putative transposable elements, 10 of which have protein coding potential. Five different transposable elements on P2 varied in nucleotide sequence between our four sequencing datasets. These differences were only a few nucleotides, and the transposable element sequences were still >99% similar. On NIES-298 P2, eight transposable elements were identical or nearly identical (eight with ≥97% identity) to transposable elements found in the NIES-298 chromosome. In general, the NIES-298 chromosome had high sequence identity to P2, driven by the presence of the transposable elements, with the longest alignment being 13,736 bp (100% identity) (Fig. S7).

### Characterization, relative transcript abundance, and analysis of Lake Erie plasmid-like and virus-like contigs

**Figure 3:**
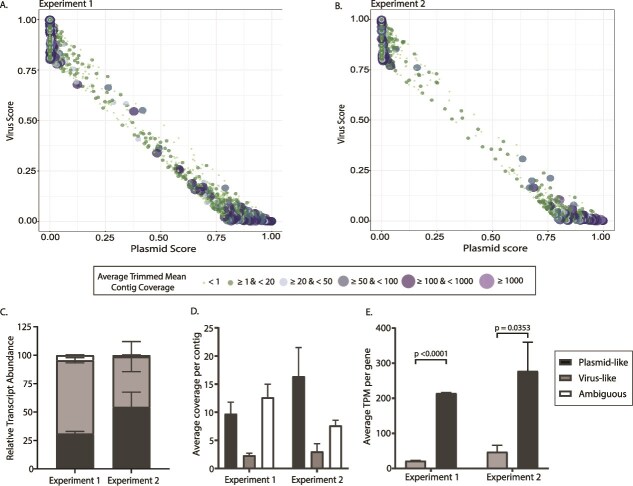
Average contig coverage, shown by dot size, and aggregated probability scores calculated from geNomad for plasmid-like, virus-like, and ambiguous contigs from experiment 1 (A) and experiment 2 (B), both experiments were done in triplicate (*n* = 3). (C) Average relative transcript abundance of virus-like, plasmid-like, and ambiguous contig groupings in experiment 1 and experiment 2. In experiment 1, virus-like contigs had the highest relative transcript abundance (avg 64.5 ± 2.29), then plasmid-like contigs (avg 31 ± 1.98), and ambiguous contigs had lowest relative transcript abundance (avg 4.5 ± 0.36). In experiment 2, plasmid-like contigs had the highest relative transcript abundance (avg 54.5 ± 13.04), followed by virus-like contigs (avg 44.3 ± 13.24). Like E1, ambiguous contigs had the lowest relative transcript abundance (1.2 ± 0.32). (D) Summarized average transcript coverage per contig in experiment 1 and experiment 2. In experiment 1, ambiguously-scored contigs had the highest average contig coverage, whereas virus-like contigs had the lowest contig coverage. In E1, “ambiguous” contigs average coverage was 12.64 (± 2.35), whereas plasmid-like contigs had average contig coverages of 9.7 (± 2.06). This contrasts with the virus-like contigs, which had average contig coverage of 2.3 (± 0.38). For experiment 2, plasmid-like contigs had the highest average contig coverage, and virus-like contigs had the lowest average contig coverage. In E2, average contig coverage for plasmid-like contigs was 16.4 (± 5.096). Ambiguous contigs had an average coverage of 7.6 (± 0.93). Virus-like contigs had lowest average contig coverages (avg 3.0 ± 1.39). (E) Average TPM per gene calculated for annotated contigs with aggregated plasmid or virus scores ≥0.9 from the experiment 1 (E1) and experiment 2 (E2) co-assemblies. Aggregated contig scores were based on the plasmid and virus output list scores by geNomad. Virus-like contigs are represented by gray bars, while plasmid-like contigs are represented by black bars. Virus-like contigs and plasmid-like contigs were not taxonomically assigned to any hosts and are representative of all sequenced phyla in the *Microcystis* bloom for E1 and E2. In both experiment 1, and experiment 2, average TPM per gene was significantly higher (FDR-adjusted *P* < .05, based on an unpaired t-test with Welch correction and Benjamini, Kreiger & Yekutieli two-stage set up) for plasmid-like contigs with scores ≥0.9 compared to virus-like contigs with scores ≥0.9.

To gain insight into the transcriptional activity of mobile elements in metatranscriptomes from *Microcystis*-dominated blooms, we characterized plasmid-like, virus-like, and "ambiguous" (geNomad scores fall between the viral and plasmid groupings) contigs in our E1 and E2 co-assemblies. For E1, a total of 22,006 contigs (out of 316,736 contigs ≥1000 bp) had chromosome scores ≤ 0.2, while E2 had 7519 contigs (out of 143,530 contigs ≥1000 bp) with chromosome scores ≤ 0.2 (Fig. 3A and B). Fig. S8 summarizes contigs in E1 and E2 that were either “virus-like”, “plasmid-like”, or “ambiguous”.

**Figure 4:**
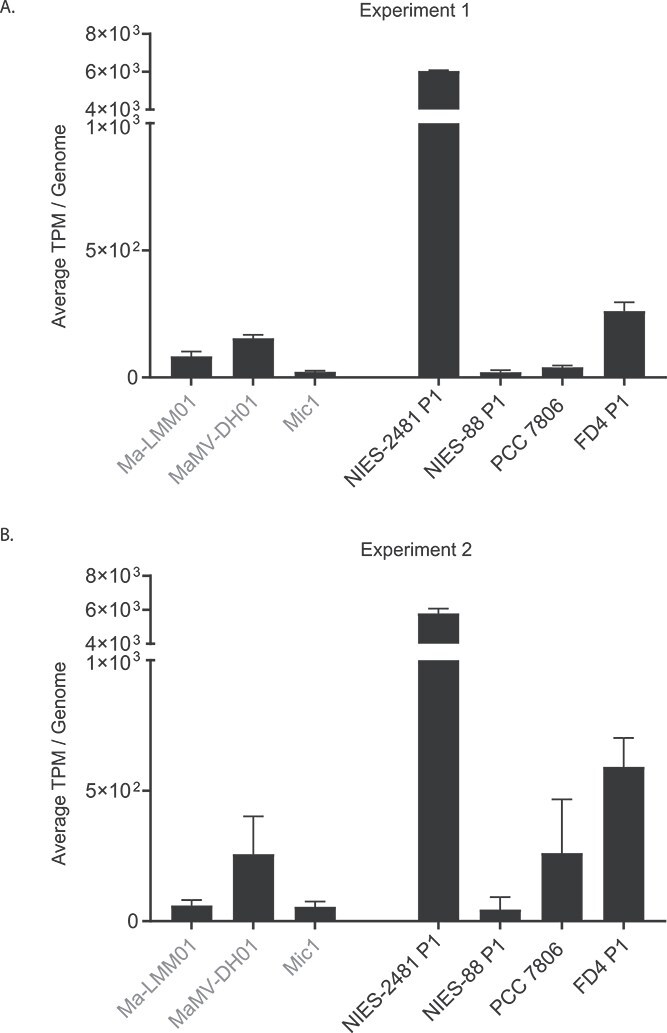
Average transcript mappings normalized by TPM (transcripts per million reads mapped) per gene for *Microcystis* phage genomes (Ma-LMM01, MaMV-DH01, Mic1, in gray font) and *Microcystis* plasmid genomes (NIES-2481 P1, NIES-88 P1, PCC 7806 (this paper) and FD4-P1, in black font) for experiment 1 (A) and experiment 2 (B). Average TPM per gene for each genome was calculated from control microcosm metatranscriptome libraries (*n* = 3) from Pound *et al.* 2022, and Martin *et al.* 2023. TPM values for all *Microcystis* phage and *Microcystis* plasmid genomes used in the analysis can be found in Table S12.

After characterizing the contigs, we calculated the relative transcript abundance of the virus-like, plasmid-like, and ambiguous contigs in the E1 and E2 co-assemblies (Fig. 3C). For E1, virus-like contigs had the highest relative transcript abundance, whereas in E2, plasmid-like contigs had the highest relative transcript abundance (Fig. 3C). However, when normalized to contig abundance, both ambiguous, and plasmid-like contigs had higher average coverage per contig then virus-like contigs in E1 and E2 (Fig. 3D).

To focus on reads that only mapped to predicted genes, we initially did an RNA-seq analysis on the viral-like, plasmid-like, and ambiguous contigs in E1 and E2 (Fig. S8). We found that in the E1 and E2 control microcosms, plasmid-like contigs had the highest average TPM per gene, followed by the ambiguous-contigs, then virus-like contigs (Fig. S8). When re-running this analysis using a more conservative subset of plasmid-like and virus-like contigs (output lists with contigs with scores ≥ 0.9), we found similar patterns upheld as our less stringent groupings, and average TPM per gene from plasmid-like contigs was significantly higher than average TPM per gene from virus-like contigs in E1 and E2 (Fig. 3E).

To investigate whether the virus and plasmid gene expression trends we observed when mapping to the E1 and E2 co-assemblies applied to *Microcystis*-specific phage and plasmid genomes, we did an RNA-seq analysis with *Microcystis*-specific plasmids and *Microcystis*-specific phage genomes available on NCBI (Fig. 4A and B). TPM values and standard deviations for all the *Microcystis* phage and plasmid genomes can be found in Table S12. Among the phage genomes we looked at, *Microcystis* phage MaMV-DH01 had the highest average TPM values in E1 and E2 (avg. TPM/gene ranged from 154–257) (Fig. 4A and B). The lowest average TPM in E1 and E2 for *Microcystis*-specific phage was Mic-1 with avg. TPM/gene values of 22–55 (Fig. 4A and B). Of the *Microcystis*-specific plasmids, six out of the 10 had average TPM/gene <20, so they were not included in Fig. 4 (values can be found in Table S12). However, four out of 10 *Microcystis* plasmids had average TPM/gene values comparable to or exceeding the *Microcystis* phage genomes. This included the NIES-2481 P1 plasmid, which had average TPM/gene values >5,000 in E1 and E2 (Fig. 4, Table S12). The FD4, NIES-88 P1, and PCC7806 plasmids also had similar average TPM/gene to the phage genomes (avg TPM ranges of 21–591) (Fig. 4, Table S12).

## Discussion

*M. aeruginosa* PCC7806 and the nontoxic Δ*mcyB* mutant have been the subject of comparative studies for more than two decades, yet the presence of a plasmid in these two strains has been underexplored. Here we show these strains maintained identical plasmids with the same copy number throughout their over 25 years in separate cultures. Additionally, all genes on the plasmid were transcriptionally active during log-phase growth in chemostats, and similar gene sequences were identified in microcosms from Lake Erie. We also found an identical nucleotide sequence to the PCC7806 plasmid on an 8,373 bp-long contig in a draft genome of *M. aeruginosa* PCC7806 (sequenced in France) from 2007, demonstrating this plasmid was not acquired in our lab. Expanding our search, we found other *Microcystis* strains which contained plasmids with DNA sequences similar or even identical to the PCC7806 plasmid. Collectively the data suggest that plasmids may be an ecophysiologically important feature of *Microcystis* strains and are stable over time and geographic distance: this is in contrast to *Microcystis* spp. genomes, which are often described as “plastic” due to abundant transposases that drive genetic rearrangements [35]. Our investigations further unveiled a phage-like plasmid in *M. aeruginosa* NIES-298, which included gene sequences consistent with a putative phage tail gene. *Microcystis* phages have been increasingly studied in recent years [36–38]. Here we demonstrated sequence overlap between *Microcystis* phage and a plasmid that could lead to “mistaken identification” of one vs the other. Collectively, our observations suggest that plasmids are an important part of the ecology of these bloom-forming cyanobacteria. Moreover, our observations create a need for caution with respect to interpretation of historically “viral” markers, which in some cases may be present on plasmids in *Microcystis* (and other) transcriptional datasets.

### Physiological relevance of *Microcystis* plasmids

In chemostat cultures, four of the PCC7806 plasmid genes [4–7, and] had comparable or higher expression values than the chromosome replication *dnaA* gene. Similarly, in the lake Erie microcosms, genes 4 and 5 were the most highly mapped to plasmid genes, with gene 4 showing TPM values ~3× higher than the microcystin biosynthesis gene, *mcyD*. Our protein model for gene 4 showed structural homology to DNA-binding proteins while gene 5 had structural homology to proteins related to the cell structure, organization, and possibly division. We hypothesize, based on our protein models, that the gene products might play a role in division/partitioning (potentially plasmid segregation/partitioning). However, this is only a hypothesis, and in the future, experimental validation will be needed to confirm the true role of these genes.

Based on our re-sequencing of the NIES-298 genome on four separate occasions, and through comparisons to other available draft genomes of NIES-298, the NIES-298 P1 plasmid also appears to be conserved. In addition, we uncovered a second and previously unreported plasmid which we refer to as “NIES-298 P2”. Unlike P1, five transposases on P2 demonstrated minor variability (still >99% identical) in nucleotide sequence between our four sequencing efforts, some of which were identical to transposases on the chromosome. While resolving transposition between the chromosome and this plasmid is beyond the present study, the observation suggests gene transfer between plasmid and chromosome may be possible. Moreover, this observation builds on the growing evidence of transposase importance in *Microcystis* [39].

### Ecological relevance of *Microcystis* spp. plasmids and their ties to phage

In our Lake Erie metatranscriptomes, we found that putative plasmid-like contigs had greater amounts of transcripts that mapped to them in comparison to putative virus-like contigs. When we mapped those same metatranscriptomes to *Microcystis*-specific phage and plasmid genomes, we saw that some *Microcystis*-specific plasmids had equivalent or higher average TPM per gene then *Microcystis*-specific phage. These observations raise new questions regarding *Microcystis* bloom ecology and the role plasmids may play, both in a broad ecological sense for HABs, and for *Microcystis* genera specifically.

Virus impacts on *Microcystis* blooms have been well documented, with the presence of phage sometimes correlated with bloom collapse [36, 38, 40]. In the current study, we found that the *M. aeruginosa* NIES-298 P1-plasmid had sequence similarity to an uncultured bacteriophage contig assembled from *in situ* sequencing data collected after a *Microcystis* bloom collapse [40]. This plasmid also demonstrated sequence similarity to a putative *Microcystis*-infecting phage genome sequence (MVGF_NODE385) from a *Microcystis* bloom collected by Morimoto *et al.* [41]: those authors demonstrated that MVGF_NODE385 was one of the most highly transcribed *Microcystis* viral genomic fragments sequenced from that bloom [41]. Studies in other microbial systems have demonstrated significant overlap between phage and plasmid in terms of their genomic content [8]. Based on NIES-298 P1, there appears to be sufficient overlap between phage and plasmid elements to warrant caution and suggest that observations in previous studies might have mistaken similar plasmids for phage [40, 41]. While several genes on NIES-298 P1 are hypothetical, our protein models point to structural homology to viral tail, DNA-binding, and capsid proteins. In the case of the putative phage tail gene on the NIES-298 P1 plasmid, it appears that this is a conserved gene found in *Microcystis* phage isolates, including Ma-LMM01. While this plasmid has some phage-like features, more work is needed to confirm the origin – this plasmid may be a phage or phage-plasmid undergoing genomic reduction, or these genes may have been acquired by horizontal gene transfer. For now, more sequencing data for *Microcystis* plasmids and phage are needed to untangle their prevalence and overlap in environmental datasets: this should include the use of long read technologies for environmental sequencing, which can help more readily obtain closed phage and plasmid sequences to help with these distinctions.

## Conclusions

This study illustrates the sequence stability as well as potential physiological and ecological relevance of *Microcystis* plasmids. *M. aeruginosa* PCC7806 has harbored a conserved plasmid in culture for >25 years, with an identical plasmid in a geographically distant *Microcystis* isolate. This conservation and the observed transcription levels suggest an important role in the cell. Moreover, the importance of plasmids may extend beyond PCC7806, as we also found a putatively conserved plasmid with phage-like features in NIES-298. Within environmental metatranscriptomes, we found that plasmids from either all phyla or *Microcystis*-specific may be important components of *Microcystis* bloom ecology and should be investigated more in future mobilome studies. Moreover, our work suggests more caution is perhaps needed in studies using short-read metagenomes or metatranscriptomes to quantify and characterize either virus or plasmid activity in the environment: future work should endeavor to provide clarity on the overlap between these mobile elements.

## Supplementary Material

Stark_et_al_2025_Supplemental_Figures_Sept_15_2025_ycaf154

Plasmid_paper_table_final_submission_ycaf154

## Data Availability

Plasmid sequences for PCC7806, NIES-298 P1 and NIES-298 P2 can be found in the GenBank database under the accessions PV068374, PV068373 and PV076616, respectively. The *M. aeruginosa*  NIES-298 host genome used in this study can be found under the accession GCA_048820285.
