# Supplementary material for: Microcystis plasmids: the unexplored portion of the mobilome and the presence of potential phage-like plasmids
Source: ISME Commun. 2025 Sep 3;5(1):ycaf154. doi: 10.1093/ismeco/ycaf154 (PMC12510456; doi:10.1093/ismeco/ycaf154)
Supplement: Stark_et_al_2025_Supplemental_Figures_Sept_15_2025_ycaf154 [file stark_et_al_2025_supplemental_figures_sept_15_2025_ycaf154.pdf]

Supplemental Figures prepared for

Microcystis plasmids: the unexplored portion of the mobilome and the presence of potential phage-like plasmids

Gwendolyn F. Stark, Laura E. Smith, Alexander R. Truchon, Robbie M. Martin, Elizabeth R. Denison, and Steven W. Wilhelm

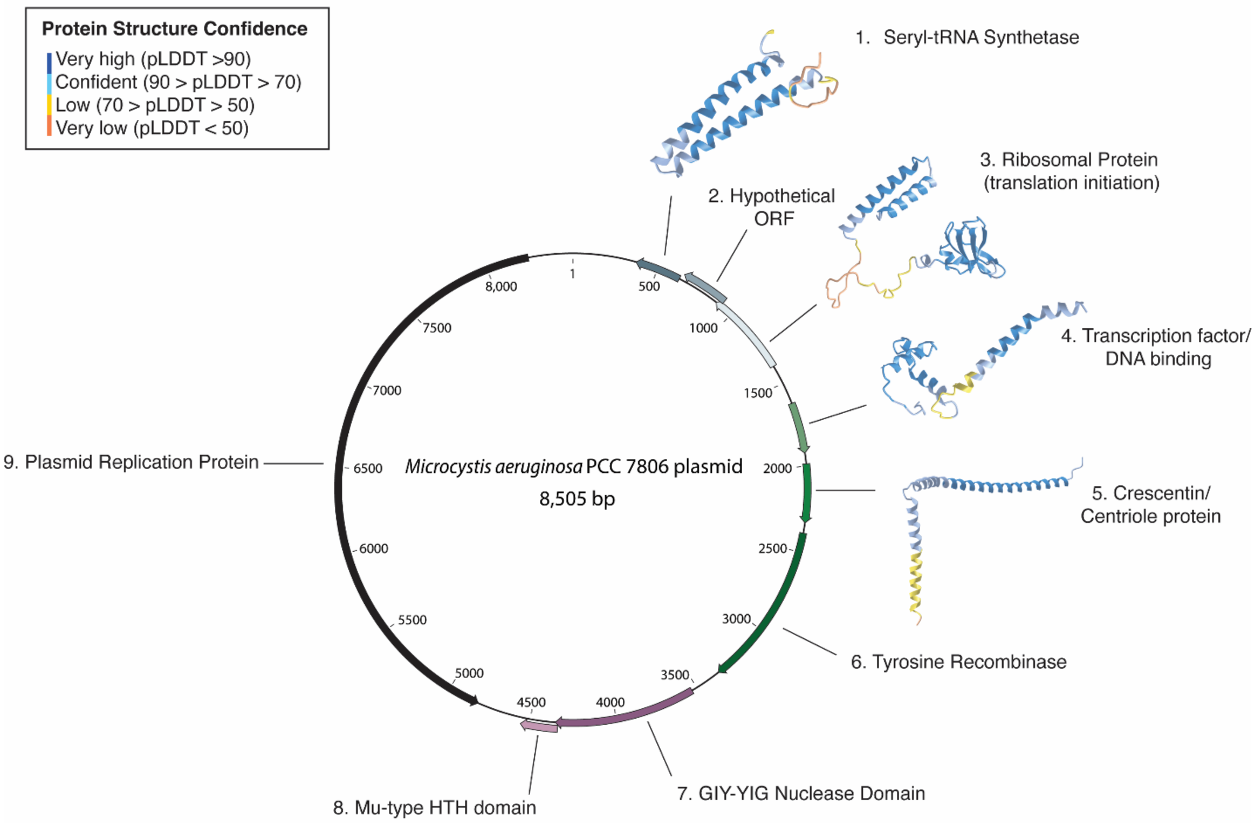

Figure S1: Plasmid found in *Microcystis aeruginosa* PCC 7806 wildtype and Δ*mcyB*. The plasmid is 8,505kb in size, and has nine predicted genes present on it, based on PGAP annotation software (Table S2). Genes 1-6 were annotated as hypothetical proteins (Table S2). Through PFAM protein domain searches and protein structure homology searches (DALI), we generated putative generalized functions of the unknown proteins (Table S3). Gene 2 had a poor protein model (pLDDT <70), and no homology matches based on our DALI search, therefore the protein structure is not shown. For gene 4, the best DALI hit (z = 5.6, RMSD = 8.1) was to a conserved putative transcription factor (pdb id: 1y9b-A). For gene 5, the best DALI hit (z = 6.7, RMSD = 17) was to a structural cell protein (pdb id: 8ahl-A). DALI outputs for genes 6-8 were similar to PFAM predictions (Tables S2, S3). DALI predicted gene 9 as an RNA helicase. OriFinder suggested a putative origin of replication occurred from residues 8248 to 370 on the plasmid.

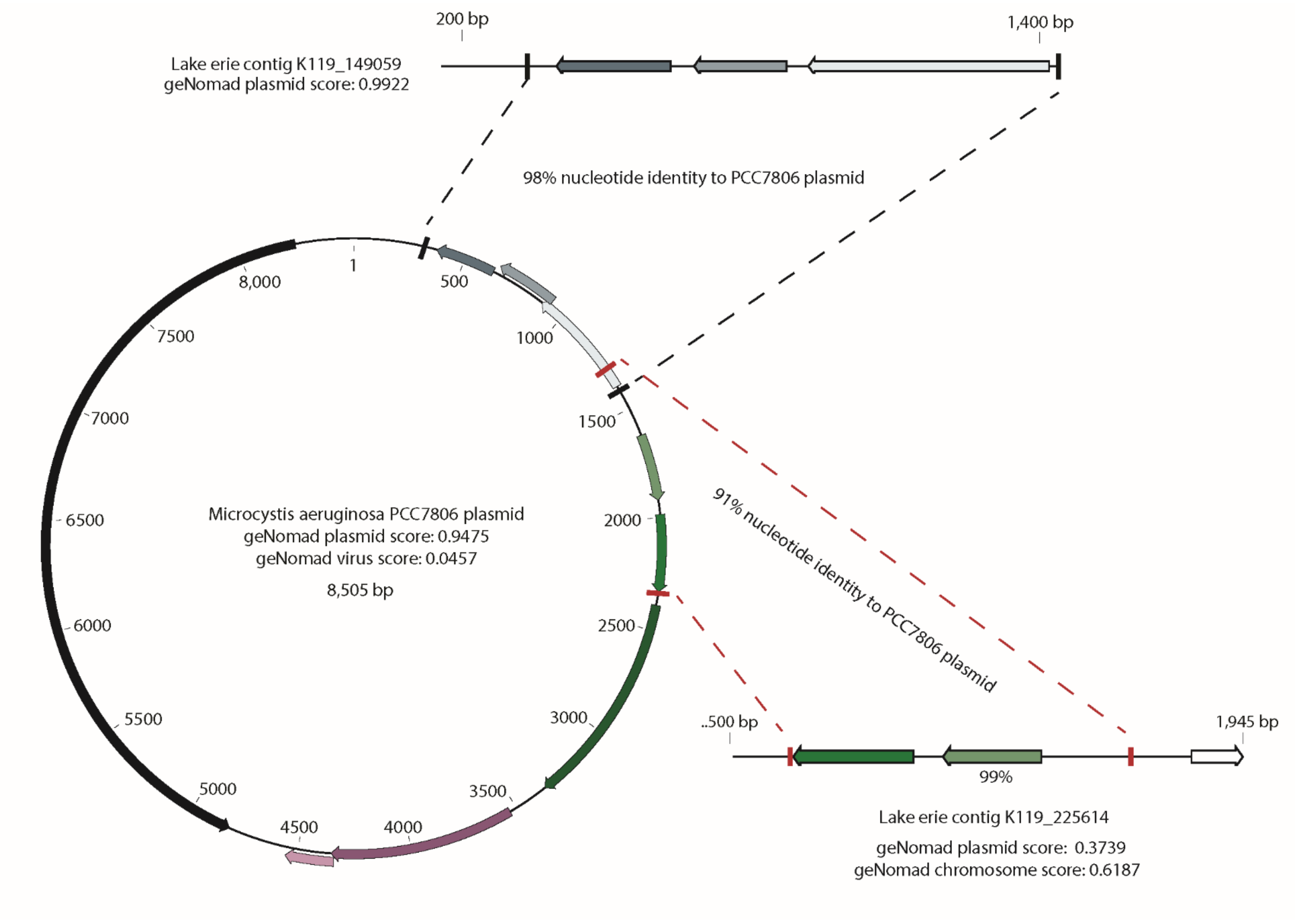

Figure S2: Contigs from Lake Erie co-assembly from Experiment 2, which had BLASTn hits with >90% nucleotide identity to the PCC 7806 plasmid.

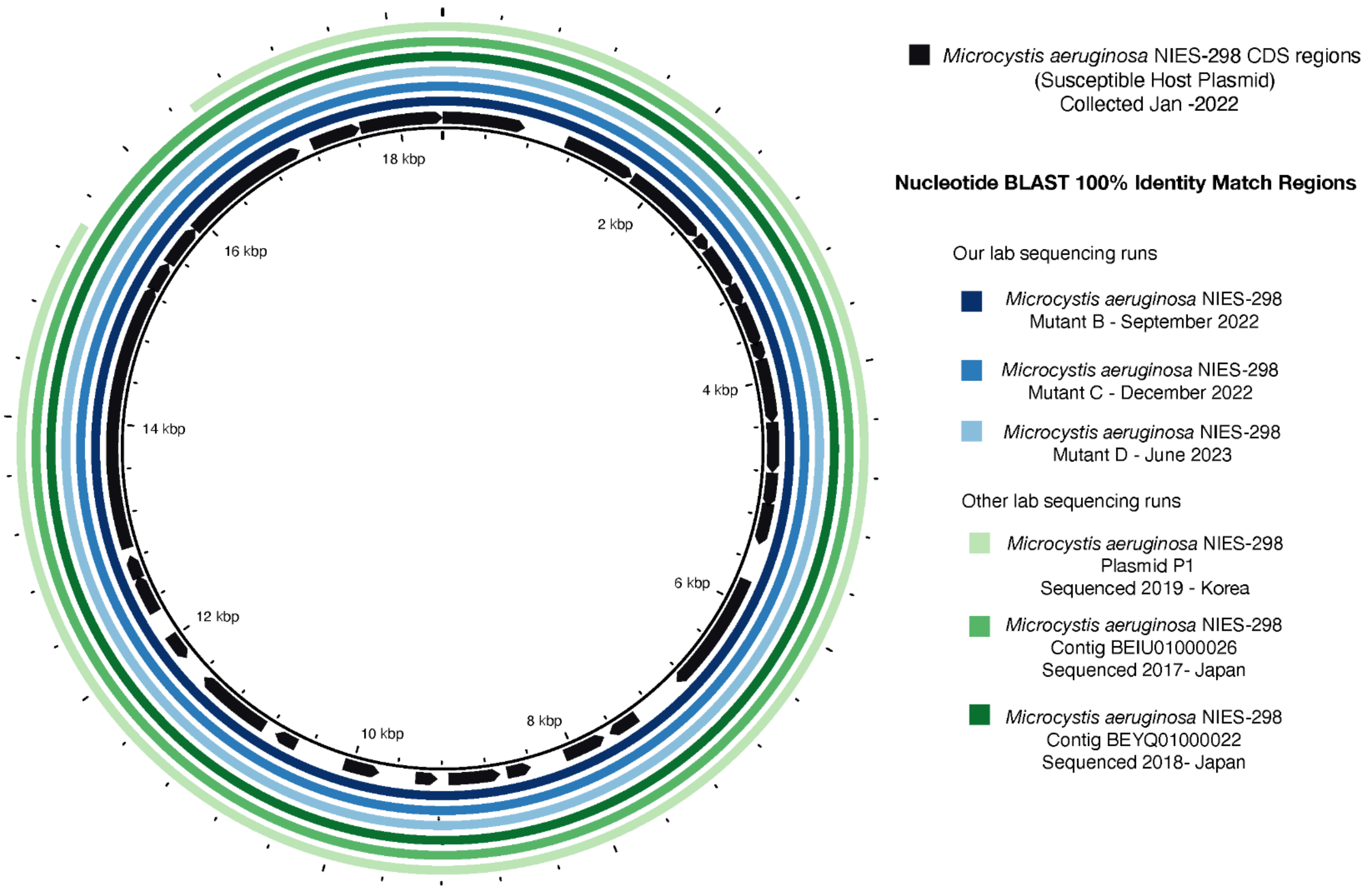

Figure S3: Nucleotide BLAST searches of a plasmid present in our *Microcystis aeruginosa* NIES-298 strain. Between our four sequencing runs (backbone coding sequences (CDS), and blue rings), the plasmid nucleotide sequences remained identical, though gene orientation on the + or – strands did change between some runs. Our BLAST of the NIES-298 plasmid against scaffold-level NIES-298 genomes (green rings) on NCBI also revealed contigs with DTR's at the ends, which were identical to the NIES-298 plasmid found in our lab sequencing runs (outside of the duplicate DTR). The NIES-298 P1 plasmid on NCBI (CP046059) is identical, aside from missing two genes (incompete light-green outer ring).

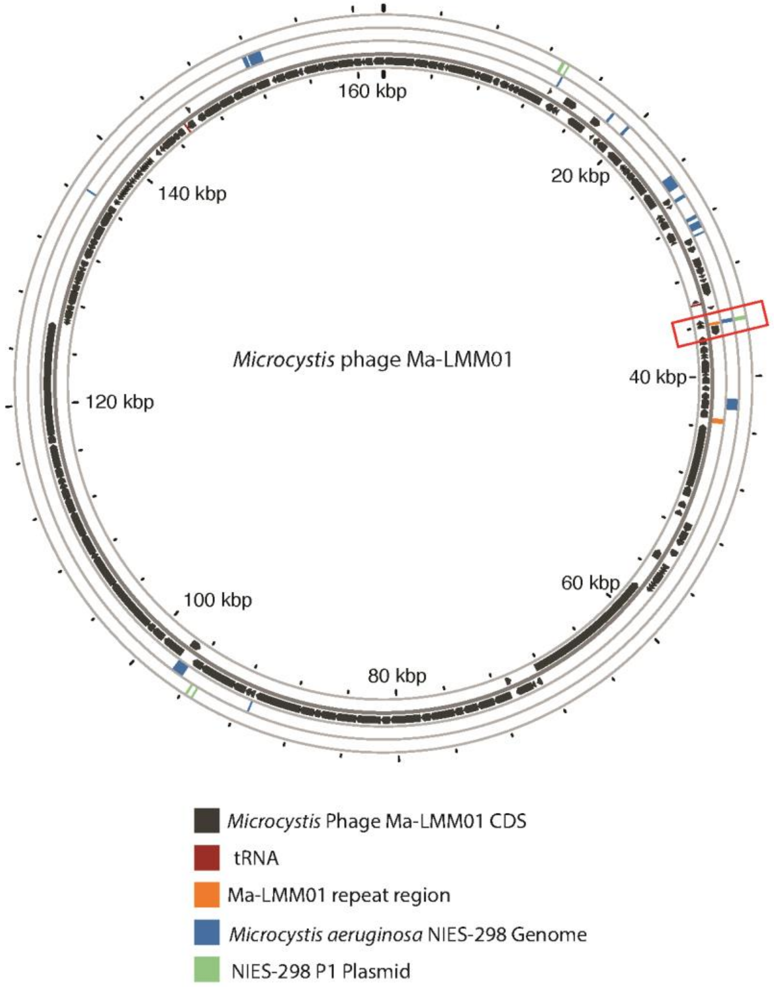


```
NIES-298 CRISPR  --GTTCCAATTAATCTTAAACCCTACTAGGGATTGAAACAAAGACC-TCTAGGAGGGCCT     57
       Ma-LMM01  CAGTTCCAATTAATCTTAAACCCTATTAGGGATTGAAAAAATAAGTGGTGCTATCTGGGCA     60
                   *********************** ************ * *     *      ** *

NIES-298 CRISPR  CAAATTCCGTTAAGAGTTCCAATTAATCTTAAACCCTACTAGGGATTGAAACCCCGTTAC     117
       Ma-LMM01  CCGACCGCATCTCTGGTTCCAATTAATCTTAAACCCTATTAGGGATTGAAAAGGGCATTT     120
                 *  *  * *     *********************** ************      *

NIES-298 CRISPR  TCATCCCTTTGCTACCCGCCCGCCTTGTTCCAATTAATCTTAAACCCTACTAGGGATTGA     177
       Ma-LMM01  ACTTATCCCTG-GGCCCCCCCGGCCTGTTCCAATTAATCTTAAACCCTATTAGGGATTGA     179
                  * *  *  **   *** **** * ************************ **********

NIES-298 CRISPR  AA-------     179
       Ma-LMM01  AAAGGGCAT     188
                 **
```

```
 Plasmid   TGTTACAATGAATCCTAACCCCTATCAGGGATTGAAAATCCAATAAGGAGTTACAGCGAT     60
Ma-LMM01   -GTTCCAATTAATCTTAAACCCTATTAGGGATTGAAAAATAAGTGGTGCTATCTGGGCAC     59
            *** **** **** *** ****** ************   * *   *   *   *  *

 Plasmid   CCTAACCCCTATCAGGGATTGAAAACATCCGTATTAACGTAAAATTGCTTTATTGGCATA     120
Ma-LMM01   -CGACCGCATCTCTGG--------------------------------------TTCCAA     80
            * * * * * ** **                                      *  *

 Plasmid   ATCAATTTAATTCCTATTAGGGATTGAAACTGATAATAGACTTAGACGGCATGACTGTCA     180
Ma-LMM01   TTAATCTTAAACCCTATTAGGGATTGAAAAGGGCATTTACTTATCCCT---------GGG     131
            * *  ****  *****************  *  * *    *    *

 Plasmid   AAATTATTACGGGTTCCAATTAATTTTAAGCCCTATTAGGGAT------ 223
Ma-LMM01   CCCCCCCGGCCTGTTCCAATTAATCTTAAACCCTATTAGGGATTGAAAA 180
                   *  ************ **** *************
```

Figure S4: Nucleotide BLAST comparison of the NIES-298 genome (blue ring regions) and the NIES-298 P1 plasmid (green ring regions) against *Microcystis* phage Ma-LMM01. The three sequences share similairty to the CRISPR repeat region in the NIES-298 genome.

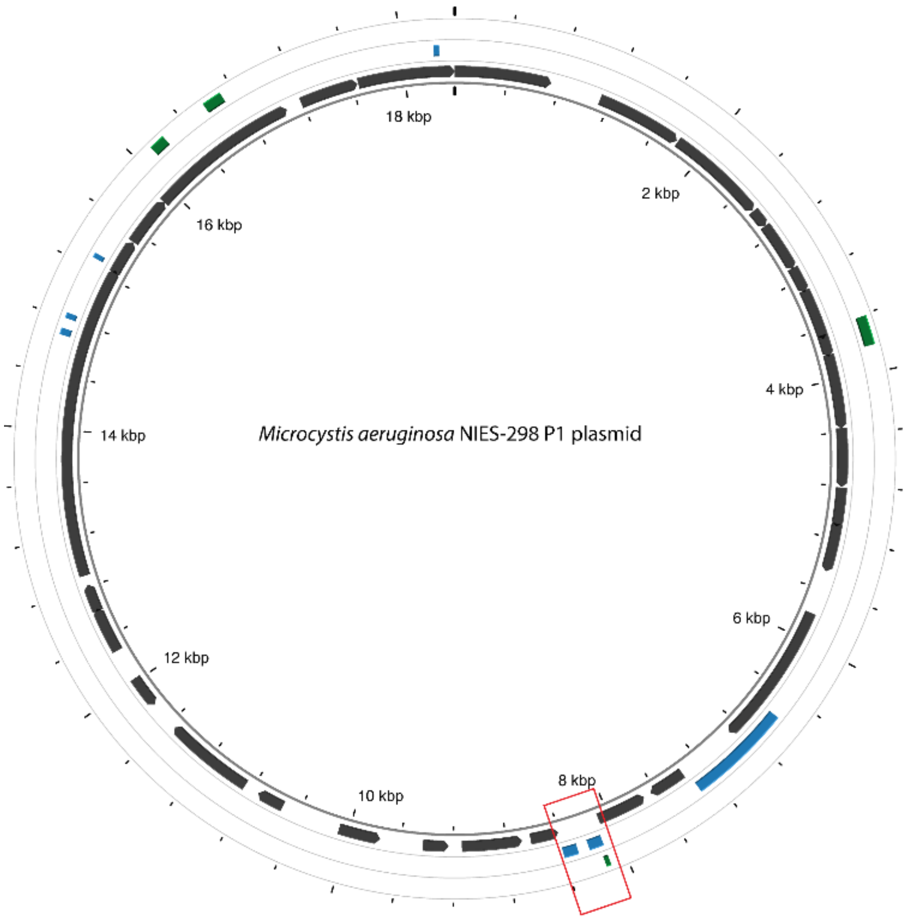

NIES-298 CRISPR & NIES-298 P1 alignment regions

```
NIES-298 CRISPR   ---ATCCCTAATAGGGTTTAAGATTAATTGGAACAATTGGATTATAG--------TCTTC   49
        Plasmid   GGCATCCCTAATAGGGCTTAAAATTAATTGGAACCCGTAATAATTTTGACAGTCATGCCG   60
                     ************* **** ************   *      *          *

NIES-298 CRISPR   TATAGCTGTGTCTATAGTTTCAATCCCTAATAGGGTTTAAGATTAATTGGAACACTTAAA   109
        Plasmid   TCTAAGTCTATTATCAGTTTCAATCCCTAATAGGAATTAAATTGATTATGCCAATAAA--   118
                  * **  * * *    *******************  ****  * * *  *   *   *

NIES-298 CRISPR   AGCATGGACGTAACAATATTAATAGGTATTGTTTCAATCCCTAATAGGGTTTAAGAT---   166
        Plasmid   -------GCAATTTTACGTTAATACGGATGTTTTCAATCCCTGATAGGGGTTAGGATCGC   171
                          *      *  ****** * **  *********** ****** *** ***
```

```
NIES-298 CRISPR   TTTCAATCCCTAATAGGGTTTAAGATTAATTGGAACGATCAATTAAAGAAGTTTTTCGCA   60
        Plasmid   TTTCAATCCCTGATAGGGGTTAGGATTCATTGTAACATGATGATCGTGGTATTTTCCCAA   60
                  *********** ****** *** **** **** ***       *   *   **** *  *

NIES-298 CRISPR   ATCCTAATAGTAGTTTCAATCCCTAATAGGGTTTAAGATTAATT   104
        Plasmid   TCATTGGGATTAGTTTCAATCCCTAATAGGGGGTAAGATTCATT   104
                      *   * *********************  ******* ***
```

NIES-298 & Microcystis phage Ma-LMM01 alignment region

```
 Plasmid   TGTTACAATGAATCCTAACCCCTATCAGGGATTGAAAATCCAATAAGGAGTTACAGCGAT   60
Ma-LMM01   -GTTCCAATTAATCTTAAACCCTATTAGGGATTGAAAAATAAGTGGTGCTATCTGGGCAC   59
            *** **** **** *** ****** ************   * *   *   *   *  *

 Plasmid   CCTAACCCCTATCAGGGATTGAAAACATCCGTATTAACGTAAAATTGCTTTATTGGCATA   120
Ma-LMM01   -CGACCGCATCTCTGG--------------------------------------TTCCAA   80
            * * * * * ** **                                        *  *

 Plasmid   ATCAATTTAATTCCTATTAGGGATTGAAACTGATAATAGACTTAGACGGCATGACTGTCA   180
Ma-LMM01   TTAATCTTAAACCCTATTAGGGATTGAAAAGGGCATTTACTTATCCCT---------GGG   131
            * *  ****  *****************  *  * *    *    *

 Plasmid   AAATTATTACGGGTTCCAATTAATTTTAAGCCCTATTAGGGAT------ 223
Ma-LMM01   CCCCCCCGGCCTGTTCCAATTAATCTTAAACCCTATTAGGGATTGAAAA 180
                    *  ************ **** *************
```

Nucleotide BLAST output regions for:

Microcystis phage Ma-LMM01 | *Microcystis aeruginosa* NIES-298 Chromosome

Figure S5: Nucleotide BLAST outputs against the NIES-298 P1 plasmid for the *Microcystis* phage Ma-LMM01 (green ring regions) and the *Microcystis aeruginosa* NIES-298 genome (blue ring regions). The only region of similarity among the three sequences was and intergenic region on the NIES-298 P1 plasmid. This region had spacer sequences similar to the NIES-298 chromosome CRISPR repeats. This was also seen for Ma-LMM01 (Figure S4).

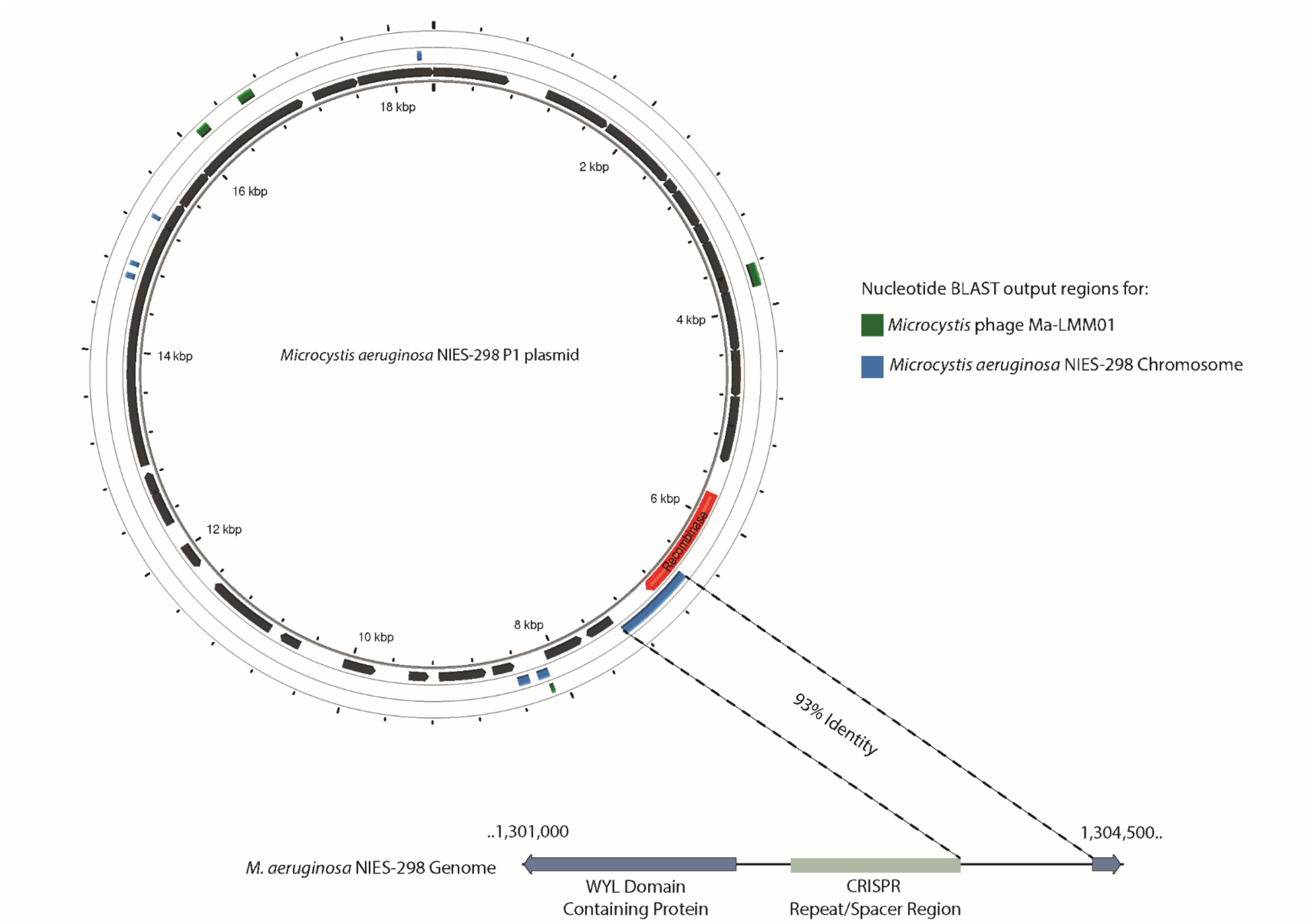

Figure S6: Two separate regions of the NIES-298 P1 plasmid have sequence similarity to CRISPR regions in the *M.aeruginosa* NIES-298 genome. Shown here, part of the tyrosine recombinase/integrase along with the intergenic region downstream is 93% identical to a region directly following a CRISPR repeat in the NIES-298 genome.

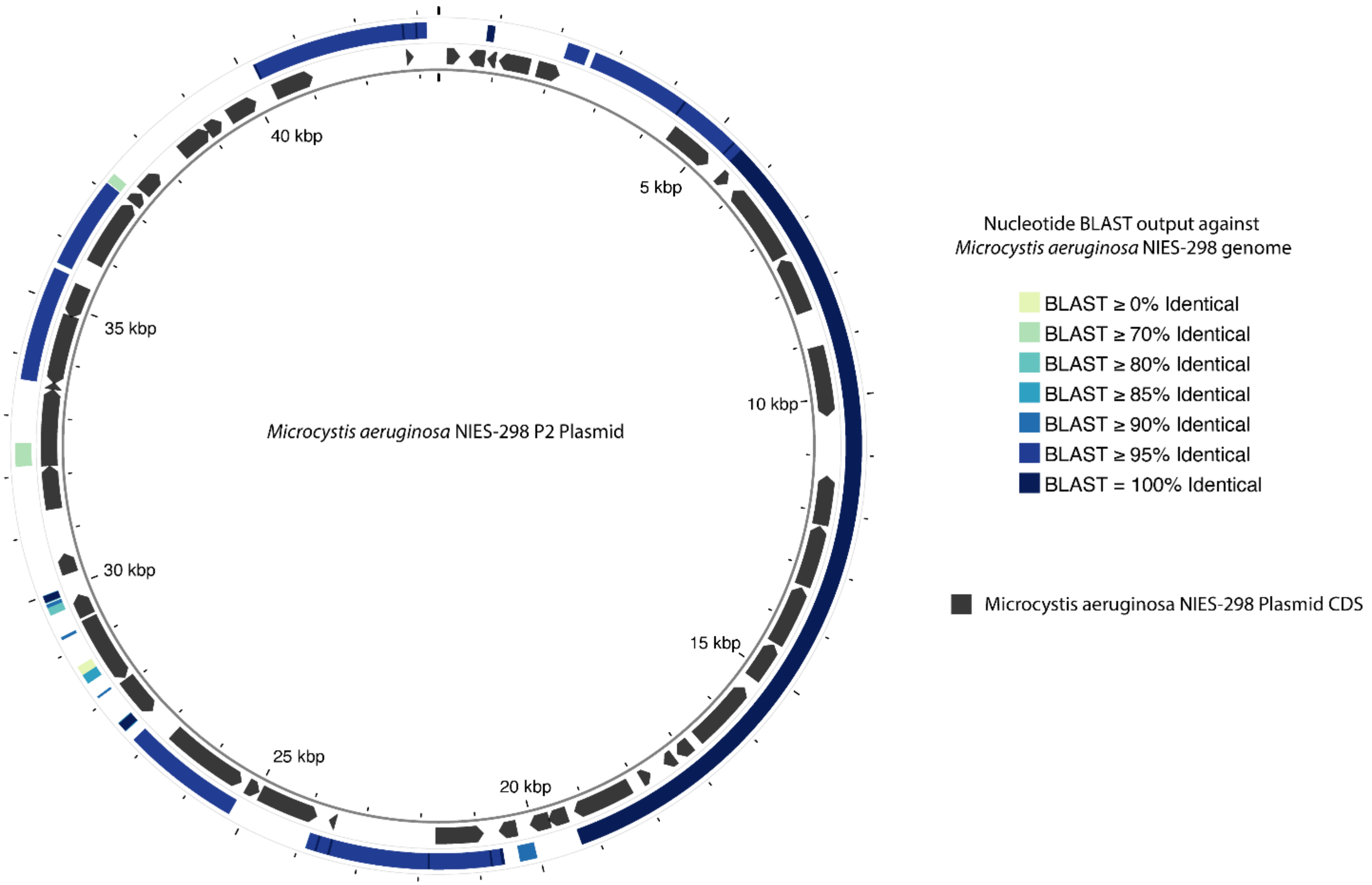

Figure S7: The *M. aeruginosa* NIES-298 P2 plasmid shares many areas of homology with the NIES-298 chromosome, with some regions being 100% identical to chromosomal regions. Many of these hits are to transposable elements in the NIES-298 chromosome.

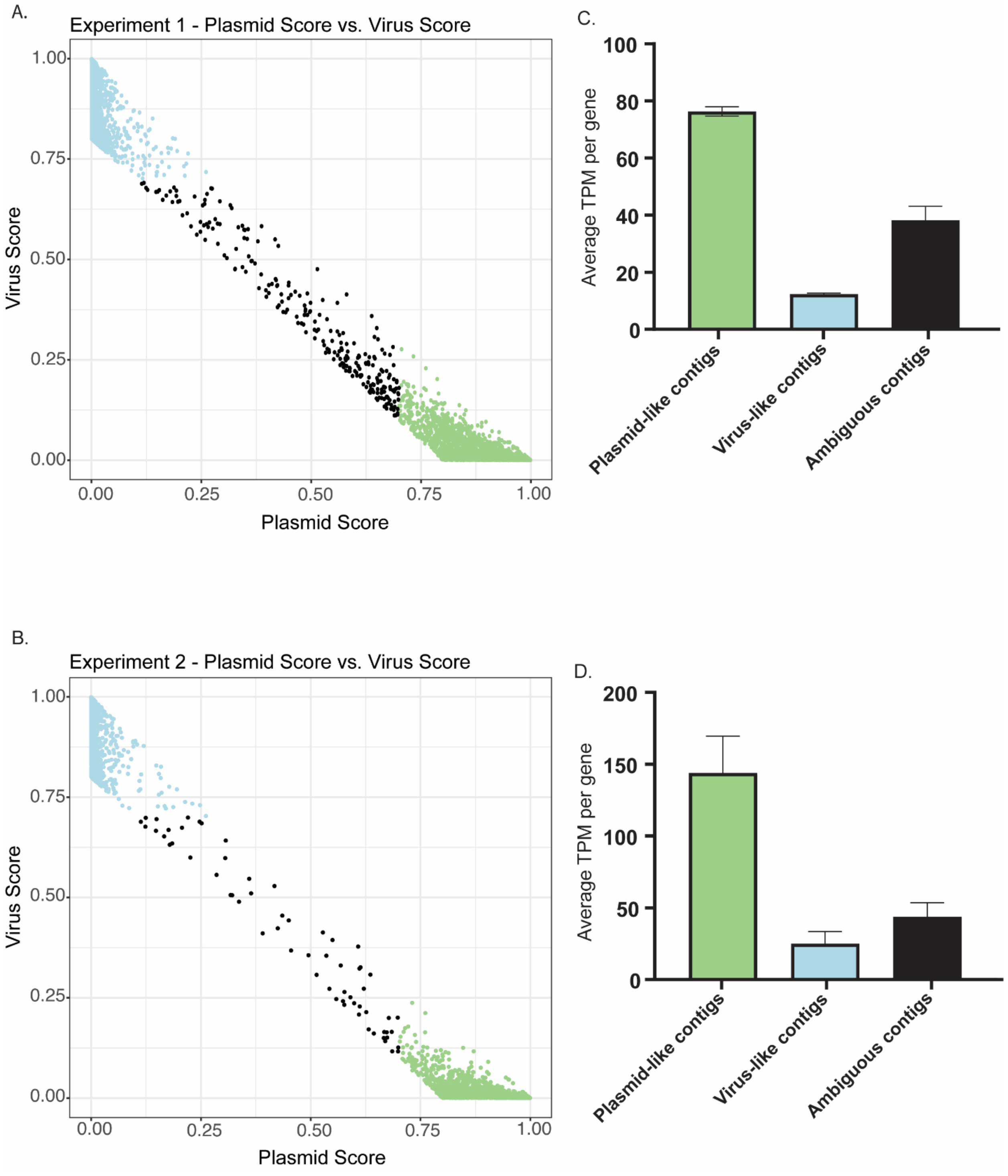
A.
Experiment 1 - Plasmid Score vs. Virus Score
Virus Score
Plasmid Score
1.00
0.75
0.50
0.25
0.00
0.00
0.25
0.50
0.75
1.00
B.
Experiment 2 - Plasmid Score vs. Virus Score
Virus Score
Plasmid Score
C.
Average TPM per gene
100
80
60
40
20
0
Plasmid-like contigs
Virus-like contigs
Ambiguous contigs
D.
Average TPM per gene
200
150
100
50
0
Plasmid-like contigs
Virus-like contigs
Ambiguous contigs

Figure S8: (A,B) Calibrated aggregated score distribution from two separate mesocosm bottle experiments from Lake Erie, “Experiment 1” and “Experiment 2”. For both experiments, n=3. Contigs were first filtered from all the contigs in the dataset based on having chromosome scores <0.2. Scores for each contigs serve as probabilities that a contig is chromosome-like, phage-like, or plasmid-like based out of a total score of 1. Dots in blue are contigs that have aggregated virus scores ≥ 0.7, in green are contigs with aggregated plasmid scores ≥0.7, and in black are contigs that have chromosome scores <0.2, but fall in a gray-area where they also have plasmid and virus scores <0.7, and therefore would not meet criteria as being more viral or plasmid-like. For E1, 19,505 contigs were “viral-like”, with virus scores ≥0.7, and 2,251 contigs were “plasmid-like” with plasmid scores ≥ 0.7 (A). 250 contigs fell outside these parameters and were considered “ambiguous” (8A). For E2, 6,109 contigs were “viral-like”, with virus scores ≥ 0.7, and 1,348 contigs were “plasmid-like” with plasmid scores ≥ 0.7 (B). Sixty-two contigs fell outside these parameters and were considered “ambiguous” (B). Gene expression analysis for Experiment 1 (C) and Experiment 2 (D), the blue-bars correspond with the viral-like contigs (scores ≥0.7), green bars correspond with the plasmid-like contigs (scores ≥0.7), and the black corresponds to the ambiguous contigs (chromosome scores <0.2, plasmid and virus scores <0.7). All metatranscriptome libraries were normalized by transcripts per million (TPM). In Experiment 1 and Experiment 2, plasmid-like contigs had the highest average TPM where as viral-like contigs had the lowest average TPM.
